# Distinct clinical characteristics of acute cardiorenal syndrome (CRS) patients: An Indian cohort study for novel biomarker discovery

**DOI:** 10.14814/phy2.70714

**Published:** 2025-12-26

**Authors:** Abhi Dutta, Sahil Verma, C. A. Athira, Ajay Bahl, Vivek Kumar, Anupam Mittal, Trayambak Basak

**Affiliations:** ^1^ School of Biosciences and Bioengineering Indian Institute of Technology (IIT) ‐ Mandi Mandi Himachal Pradesh India; ^2^ Department of Translational and Regenerative Medicine Postgraduate Institute of Medical Education and Research Chandigarh India; ^3^ Department of Cardiology Postgraduate Institute of Medical Education and Research Chandigarh India; ^4^ Department of Nephrology Postgraduate Institute of Medical Education and Research Chandigarh India

**Keywords:** acute kidney injury, biomarker, cardiorenal syndrome, cohort study, heart failure

## Abstract

The growing prevalence of cardiorenal syndrome (CRS) worldwide underscores the need for specific clinical management, diagnosis, and identification of CRS‐specific novel biomarkers. Acute‐CRS is highly prevalent in Acute Heart Failure (AHF) patients, clinically driving adverse outcomes. However, there is a paucity of clinical data on the overlap between CRS, HF, and acute kidney injury (AKI). Pathophysiologically, acute‐CRS begins with an acute myocardial injury, which subsequently leads to renal insufficiency. Clinically, reduced ejection fraction (EF), increased creatinine(sCr), and reduced eGFR are the standard parameters for diagnosis and treatment of acute‐CRS. To understand the clinical need, this study compares the clinical characteristics of the acute‐CRS patients with CHF, AKI, and healthy. Recruited participants were of Indian origin, diagnosed with any one of CRS‐I, HF, or AKI, or healthy individuals. Baseline demographics were compared across groups and further subjected to clustering analyses. EF, eGFR, and sCr varied significantly across all groups in the cohort. Clustering analysis shows a distinct pathological grouping of Acute‐CRS. Intriguing differences in eGFR and EF exhibit sex bias in clinical Acute‐CRS cases. Acute‐CRS is also clinically distinct in terms of clinical biomarkers of AKI. Hence, this cross‐sectional cohort study establishes acute‐CRS as a distinct pathological condition requiring comprehensive studies.

## Graphical abstract

Characteristic distinction of Acute Cardiorenal Syndrome (CRS‐1) in clinics: Insights from an Indian Cohort.

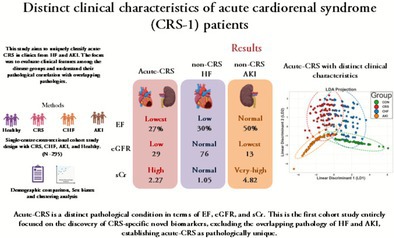

## 1 INTRODUCTION

Cardiorenal Syndrome (CRS) encompasses a common pathophysiological dysregulation resulting in the simultaneous co‐occurrence of cardiac and renal dysfunctions (Rangaswami et al., 2019; Ronco et al., 2008). The Acute Dialysis Quality Initiative (ADQI) consensus in 2008 introduced the first classification system for CRS into five distinct clinical subtypes (Ronco et al., 2008). Clinically, CRS encompasses variable grades of cardiorenal insufficiencies classified into five clinically distinct subtypes (Rangaswami et al., 2019). Of them, acute CRS is the most prevalent in clinical settings (Dutta et al., 2023). In India, Acute Cardiorenal Syndrome (CRS) is studied as the most prevalent form of CRS, affecting individuals across all age groups (Prothasis et al., 2020; Vernooij et al., 2024). The prevalence of Cardiorenal syndrome differs from 25% to 40% globally, indicating its extensive influence on clinically admitted heart failure (HF) patients (Athwani et al., 2017; Dutta et al., 2023; Prothasis et al., 2020; Uduman, 2018). The prevalence is particularly high in India, where studies show that about 30% of individuals with heart failure also have CRS, highlighting the need for an optimized treatment approach in the area (Dutta et al., 2023; Shah et al., 2016).

**FIGURE 1:**
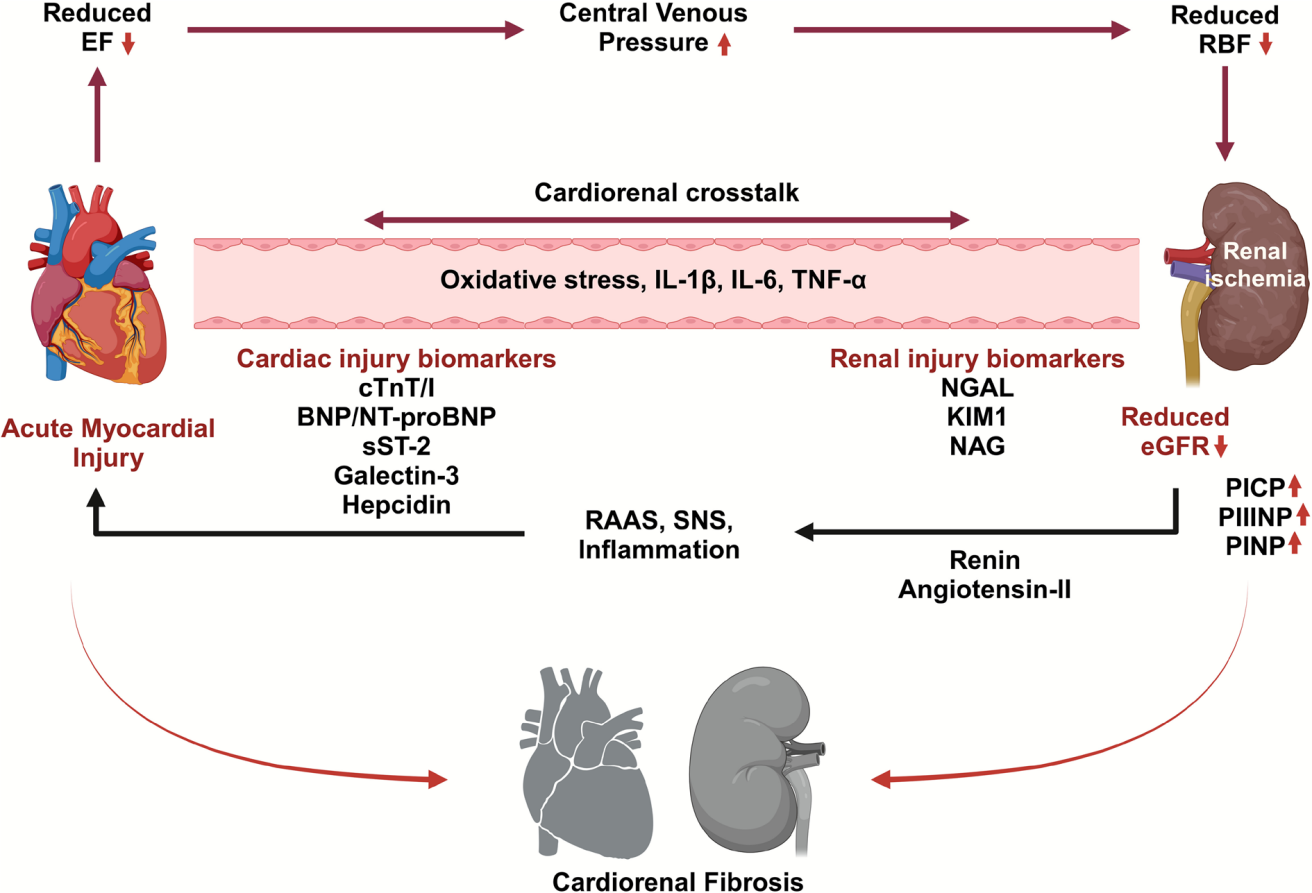
Pathophysiology of acute CRS. Acute myocardial injury causes a significant drop in ejection fraction (EF), which eventually induces a sudden increase in central venous pressure (CVP). Low EF with increased CVP causes renal hypoperfusion, triggering renal ischemia. Ischemic renal injury, in turn, activates the renin‐angiotensin‐aldosterone‐system (RAAS), the sympathetic nervous system (SNS), and inflammatory pathways. A significant impact of such pathologic cardiorenal crosstalk ends up in cardiorenal fibrosis and organ failure in CRS. Created in BioRender. Dutta et al. (2025) https://BioRender.com/g96p595.

Hence, it is of foremost priority to understand the broad pathophysiology of the clinical outcomes observed in acute CRS. Acute heart failure (AHF) causes a rapid decline in left ventricular ejection fraction (LVEF), leading to increased central venous pressure and decreased tissue perfusion (Figure 1) (Dutta et al., 2025; Mitsas et al., 2022). Cardiorenal crosstalk in acute CRS, starting with acute compensation of cardiac functioning, triggers an ischemic injury in the kidney due to reduced renal blood flow and hypoperfusion (Cruz, 2013; Rangaswami et al., 2019). Renal hypoperfusion triggers oxidative stress and a sudden onset of ischemia‐induced acute kidney injury (AKI). AKI, underlined with acute cardiac dysfunction, potentially upregulates the neurohormonal response through activation of the Renin‐Angiotensin‐Aldosterone‐System (RAAS), Sympathetic Nervous System (SNS), Natriuretic Peptide Systems (NPS) associated with increased oxidative stress and inflammatory responses (Cruz, 2013; Dutta et al., 2023). Such an onset of complex and multifactorial neurohormonal pathways establishes the pathological condition of acute CRS (Rangaswami et al., 2019; Ronco et al., 2008; Zannad & Rossignol, 2018). However, a varied level of renal dysfunction exists in clinical acute CRS patients and has pronounced detrimental effects on the pre‐dysfunctional myocardium (Fedele et al., 2017; Seckinger et al., 2022). A notable overlap of pathological phenotype with HF and AKI complicates clinical diagnosis, management, and outcomes, especially in elderly patients with comorbidities, where it gets further complicated (Pradhan et al., 2022; Reddy et al., 2020; Shah et al., 2016; Tandon et al., 2013).

Recent clinical studies have extensively reported a significant rise in CRS prevalence associated with increased circulatory plasma markers of HF and AKI, but the multifactorial pathophysiology (Figure 1) necessitates CRS‐specific biomarkers. A persistent problem with clinical management of CRS in AHF is delayed diagnosis (Rangaswami et al., 2019; Zannad & Rossignol, 2018). Managing HF patients in the presence of AKI is challenging since it significantly raises adverse clinical outcomes (Chen et al., 2022; Hobson et al., 2009). AKI is the only independent predictor of mortality both within 1 year and in the long term for patients with acute HF (Chen et al., 2022; Moore et al., 2018; Rademaker et al., 2021). From the pathological perspective, CRS is a multifactorial disease, and the pathophysiology describes it as a continuum of cardiac and renal dysfunction mediated through hemodynamic, inflammatory, and fibrogenic pathways (Dutta et al., 2023; Rangaswami et al., 2019; Zannad & Rossignol, 2018). Individualistic treatment of kidney insult or heart failure is the current clinical practice in the management of CRS (Rangaswami et al., 2019; Ronco et al., 2010). However, considering the multivariant pathogenesis, the major constraint in CRS clinical management is the diagnosis and prognosis of the disease pathogenesis (Dutta et al., 2023). Several means of diagnostics, including non‐invasive imaging and hemodynamic monitoring, are currently being used alongside biomarker evaluations (Rangaswami et al., 2019; Ronco et al., 2008). HF‐associated markers such as Troponin T/I, Brain Natriuretic Peptide (BNP)/N‐terminal pro‐BNP (NT‐pro‐BNP), Soluble suppression of tumorigenicity 2 (sST‐2), Galectin‐3, Hepcidin, and novel AKI‐associated biomarkers, including Kidney Injury Molecule (KIM)‐1, N‐acetyl‐beta‐D‐glucosaminidase (NAG), and serum Neutrophil Gelatinase‐Associated Lipocalin (NGAL) are broadly used to diagnose AKI and HF in CRS (Chung et al., 2022; Dutta et al., 2023; Endre & Pickering, 2014; Ricci et al., 2011; Sabbisetti et al., 2014; Zannad & Rossignol, 2018). Although cardiac and renal biomarkers help provide information regarding the pathological condition, unique diagnostic biomarkers remain elusive to date (Cruz, 2013). None of the presently diagnostic or prognostic markers is specific to acute CRS (Dutta et al., 2023).

The cohort developed in this study will enable us to explore CRS‐specific biomarkers and their pathophysiological impact on the syndrome. The purpose of this study is to build a cross‐sectional cohort to describe clinical etiologies of acute CRS in correspondence with HF and AKI. We also investigated the clinical and hematological changes in acute CRS and compared them with those observed in individuals with HF, AKI, and healthy controls. The intended inclusion of two groups, that is, HF and AKI, has also led to the unbiased characterization and stratification of acute CRS‐related clinical parameters.

## 2 MATERIALS AND METHODS

### 2.1 Study design

This is a cohort investigation designed to study the gross changes of heart and kidney functioning in an Indian cohort of acute CRS. The cohort we have designed in a way so that it could uniquely study the pathophysiology and biomarker assessment of acute CRS, while rejecting all overlapping features of HF and AKI. To achieve this, we have included four groups in our biomarker cohort between 2023 and 25: (1) CRS (Type 1), (2) AKI (Without CRS symptoms), (3) Chronic heart failure (CHF, without CRS symptoms), and (4) Healthy (without any HF, AKI, or CRS etiologies). All recruitment was done following guidelines for HF and AKI. The records of all 295 patients, including three different etiologies and healthy individuals, are initially recorded in the Cardiology and Nephrology clinics at the Postgraduate Institute of Medical Education and Research (PGIMER), Chandigarh, India.

The future prospect of this study is planned into two phases:

Phase I is the discovery of biomarkers specific to acute CRS. Patient recruitment for this phase was conducted in groups of 10 patients from each of the CRS, CHF, AKI, and healthy groups. The plasma samples were collected and studied for OMICS‐based identification of novel CRS‐specific biomarkers.

Phase II will be the Replication (validation) phase, with 40 patients with CRS‐I, 40 patients each with CHF and AKI, and age‐sex matched controls. Unique CRS‐OMICS candidates will be validated on a large scale for biological relevance and robustness of the study.

## 3 PATIENT RECRUITMENT

All study subjects were recruited from the Department of Cardiology and Nephrology Clinics at the Postgraduate Institute of Medical Education and Research, Chandigarh, with written informed consent. This study was approved by the Institutional Ethics Committee of the PGI‐Chandigarh (PGI/IEC/2022/000870) and IIT‐Mandi (IITM/IEC(H)/2022/TB/P3). Baseline medical and laboratory data were successfully collected from all 295 subjects and will be used for further analysis of findings from this cohort. Patients with heart failure (HF), as defined by the European Society of Cardiology (ESC) and Heart Failure Association (HFA), were extensively screened for enrollment between April 2023 and June 2025. The study already included a total of 91 CRS patients, 88 CHF patients and 64 AKI patients. Additionally, 52 healthy individuals who visited the health center for reasons unrelated to CRS, HF, or AKI were included. Clinical characteristics, inclusion, and exclusion criteria of each patient group are tabulated in Table 1.

**TABLE 1:** Demographic data and clinical features of the cohort.

Characteristics	CON	CRS	CHF	AKI
Total patients, *n*	52	91	88	64
Male, *n*	32	61	57	37
Female, *n*	20	30	31	27
Mean age, SEM	48.4 ± 2.2	61.90 ± 1.6	57.08 ± 1.74	38.37 ± 1.82
Heart failure	No	Yes	Yes	No
HFpEF, *n*	‐	7	14	‐
HFmrEF, *n*	‐	14	16	‐
HFrEF, *n*	‐	70	58	‐
Acute kidney injury	No	Yes	No	Yes
Risk, *n*	‐	11	‐	4
Injury, *n*	‐	57	‐	9
Failure, *n*	‐	23	‐	51
Ejection fraction (EF, %)^a^	54.32 ± 2.88	27 ± 10.09	30.34 ± 11.59	50 ± 0.62
HFpEF	‐	50 ± 0	50.61 ± 1.59	‐
HFmrEF	‐	40.34 ± 1.29	41.45 ± 2.12	‐
HFrEF	‐	23.4 ± 6.29	24.6 ± 7.38	‐
eGFR (mL/min/1.73 m^2^)^a^	95.44 ± 32.86	29.05 ± 14.98	76.26 ± 22.86	12.76 ± 11.88
Risk	‐	58.68 ± 12.1	‐	49.49 ± 1.12
Injury	‐	34.26 ± 6.68	‐	29.8 ± 5.09
Failure	‐	13.8 ± 5.45	‐	9.87 ± 4.8
Creatinine (mg/dL)^a^	0.77 ± 0.3	2.27 ± 1.43	1.05 ± 0.034	4.82 ± 2.81
Risk	‐	1.39 ± 0.28	‐	1.55 ± 0.09
Injury	‐	1.97 ± 0.27	‐	2.61 ± 0.42
Failure	‐	4.06 ± 1.77	‐	5.86 ± 2.55
Plasma NGAL (ng/mL)^b^ (mean ± SEM)	130 ± 30.48	334.54 ± 65.38	‐	1316.55 ± 242.03
Plasma KIM‐1 (pg/mL)^b^ (mean ± SEM)	Not detected (*n* = 14) Not Detected	272.34 ± 412.41 (*n* = 26)	‐	606.7 ± 195.37 (*n* = 26)
Alcohol	4%	13.80%	15.50%	0%
Hypertension	11.50%	41.40%	43.30%	10.70%
Diabetes	13%	40.20%	36.60%	7.60%
Smoking	0%	19.14%	23%	0%
Sodium/Potassium (mmol/L)	‐	135.3/4.3	135.07/4.1	‐
Human ferritin (ng/mL)	58.74	58.4	170.12	200.08
NYHA class	‐	III/IV	III/IV	‐
Ischemic/Non‐ischemic	‐	49/42	74/14	‐
Common medications				
Diabetes	Metformins, sulfonylureas, and thiazolidinediones			
Hypertension	Amlodipine, ACE inhibitors, and Angiotensin receptor blockers (ARBs)			

Abbreviations: AKIP, acute kidney injury; CHF, chronic heart failure; CON, control; CRS‐I, cardiorenal syndrome type I; HFmrEF, HF with mildly reduced EF; HFpEF, HF with preserved EF; HFrEF, HF with reduced EF; KIM‐1, kidney injury molecule 1; NGAL, neutrophil gelatinase‐associated lipocalin; NYHA, New York Heart Association; SEM, standard error of mean.

^a^ Values are presented as mean ± standard deviation (SD).

^b^ Age‐sex matched.

## 4 PATIENT DETAILS

Patients with cardiovascular disease of any etiology who had an in‐hospital stay longer than 24 h, with or without cardiorenal dysfunction, were included in the study. Patients included preferentially had an HF condition of New York Heart Association classes (NYHA) 3–4 or AKI as classified with RIFLE (Risk, Injury, Failure, Loss of kidney function, and End‐stage kidney disease). Patients in Group III (CHF) have serum creatinine (sCr) levels less than 1.5 mg/dL, EF <50%, and comorbidities related to heart failure (i.e., hypertension and diabetes). Patients who satisfied all of the requirements of Group III but had acute kidney injury (serum creatinine level higher than 1.5 mg/dL) were included in Group II, or the CRS group. Patients with AKI having a sCr level of >1.5 mg/dL and a Glomerular filtration rate (GFR) of <60 mL/min/1.73 m^2^ with a normal cardiac EF of above 50% were enrolled in Group IV, which was designated as the acute kidney injury (AKI) group. Further, the AKI diagnosis was based on baseline elevated sCr levels (following the KDIGO guidelines), and any other underlying preexisting kidney dysfunctions were excluded clinically by the Nephrologist at PGIMER, Chandigarh (A tertiary clinical centre). Patients with normal cardiac EF (above 50%) and sCr levels (below 1.5 mg/dL), who also had concomitant conditions including diabetes and hypertension, made up Group I, the control group.

## 5 SAMPLE AND DEMOGRAPHIC PARAMETERS

Patients' plasma was collected from venous blood samples. Alongside, vital parameters were also noted at the time of patient recruitment with consent. Routine laboratory measurements were performed by expert clinical technicians at the point of recruitment. Quantitative measurements of creatinine levels were done by kinetic colorimetric assay using the Creatinine Jaffe Gen.2 (Ref no. 06407137214) kit from Roche. eGFR is calculated by the Cockroft‐Gault Formula. Neutrophil gelatinase‐associated lipocalin (NGAL) (Genlisa Human NGAL ELISA Kit, Krishgen Biosystems, range 0.156–10 ng/mL, Ref. KBH3872) and Kidney Injury Molecule (KIM‐1) (Genlisa Human KIM1 ELISA Kit, Krishgen Biosystems, range 78–5000 pg/mL, Ref. KBH7067) were measured with collected plasma samples from patients.

Echocardiographic assessments were done for cardiac functionalities by a professional healthcare technician at the primary care setup using a clinical ultrasound system. Ejection fraction (EF) is calculated by dividing the stroke volume (SV) by the end‐diastolic volume (EDV) and multiplying by 100.

## 6 LINEAR DISCRIMINANT ANALYSIS (LDA)

To further examine how discriminant the disease groups in our cohort are, we have used LDA as a supervised clustering method to study the sample group relationships in terms of EF, eGFR, and sCr. Patient IDs were labeled with respective pathological conditions, and the model was trained to assess group spread and overlap in the LDA plot. Ellipses represent the 95% confidence interval. All analyses were done using Python in the Visual Studio Code (VS Code).

## 7 STATISTICAL ANALYSIS

The total sample size was determined using the software *ClinCalc* (Kousa et al., 2024). Statistical analysis was performed using GraphPad Prism 9.0. Statistical significance between 3 or more groups was analyzed with one‐way ordinary ANOVA followed by post‐hoc multiple comparisons with Tukey's test. For non‐Gaussian data, significance in two groups was assessed with the Mann–Whitney or Kruskal–Wallis test. The Student's *t*‐test for unpaired samples was applied to determine statistical differences between two groups. A *p*‐value of ≤0.05*, ≤0.01**, ≤0.001***, and ≤0.0001**** was considered statistically significant.

## 8 RESULTS

### 8.1 Clinical characteristics of the cohort

The overall clinical demographics and cohort details are summarized and tabulated in Table 1. All the patient data are recorded at the time of their last clinical attendance.

### 8.2 Group I‐Control

This group consists of a total of 52 individuals visiting the clinic with etiologies other than HF, AKI, or CRS and had no symptoms of any of the disease pathologies overlapping with the three other groups of this cohort. The cardiac functionalities were normal as indicated by the mean EF (%) of 54.32 ± 2.88. Two surrogates of renal dysfunction also showed up to be normal with an average sCr level at 0.77 ± 0.3 mg/dL and an eGFR of 95.44 ± 32.86 mL/min/1.73 m^2^. We also measured the plasma ferritin level, which stood at 58.74 ng/mL. However, due to the lack of any considerable pathological symptoms, Sodium/Potassium couldn't be tested in this group. Mean levels of plasma NGAL were significantly lower than those of AKI, and undetected KIM‐1 levels signify a healthy group of controls without any AKI, HF, or CRS‐related aetiologies.

### 8.3 Group II‐CRS‐I

At the time of the last record, among patients visiting the clinics for CRS‐related symptoms, 91 patients had pathologies that met our inclusion criteria. All the patients had a compromised EF (%) with sCr levels considerably above the baseline value. A severely compensated estimated GFR with a mean of 29.05 ± 14.98 mL/min/1.73 m^2^ is also recorded to be substantially below the normal level. Hypertension and diabetes were clinically diagnosed in 38 (41.4%) and 36 (40%) individuals, respectively. Plasma Sodium/Potassium levels were 135.3/4.3 mmol/L. The circulating level of ferritin was 58.4 ng/mL in this group. Patients were classified as NYHA Class III or IV. Additionally, there were 49 patients with ischemic etiology of AHF and 42 patients with non‐ischemic heart failure.

### 8.4 Group III–CHF

Among the individuals, patients without clinical renal insufficiency and with AHF‐related etiologies, a total of 88 patients who had met our inclusion criteria were included in the CHF group. A severely compromised EF (%) signifies severely compromised cardiac functionality typical of HF. Among the patients, there were 14 (16%) patients with preserved EF, 16 with mildly reduced EF (18.1%), and 58 (65.9%) patients who had reduced EF. At admission, sCr values were normal, and eGFR was also within the normal range. Clinical symptoms of hypertension and diabetes were present in 38 (43.3%) and 32 (36.6%) patients, respectively. Plasma Sodium/Potassium levels were 135.07/4.1 mmol/L. We have also recorded that the circulating level of ferritin, a surrogate for iron level, was 170.12 ng/mL in this group. All participants clinically presented with NYHA Class III and IV. Additionally, there were 74 patients with ischemic etiology of AHF and 14 patients with non‐ischemic HF, like DCMP.

### 8.5 Group IV‐AKI

Patients visiting nephrology clinics with potential symptoms of acute renal dysfunction were evaluated for AKI, and 64 individuals met the inclusion criteria, forming the AKI group. The group exhibited a severely reduced eGFR with elevated sCr levels, indicating significant renal dysfunction (Table 1). Despite these renal impairments, all individuals had a normal cardiac function, as evidenced by a normal EF, effectively ruling out overlap with Group II (CRS type 1) of the cohort. Due to a lack of clinical necessities, Sodium/Potassium wasn't tested in this group. Furthermore, we found that the AKI group had a significantly elevated mean plasma level of NGAL 1316.55 ng/mL compared to 130 ng/mL in controls and 334.54 ng/mL in CRS patients (Table 1). Furthermore, plasma KIM‐1 level was not detected in the healthy controls evaluated. We also found that the AKI group had a significantly elevated mean plasma level of KIM‐1, 606.7 pg/mL compared to 272.3 pg/mL in CRS patients (Table 1).

### 8.6 Global differences in baseline demographic features in the cohort

**FIGURE 2:**
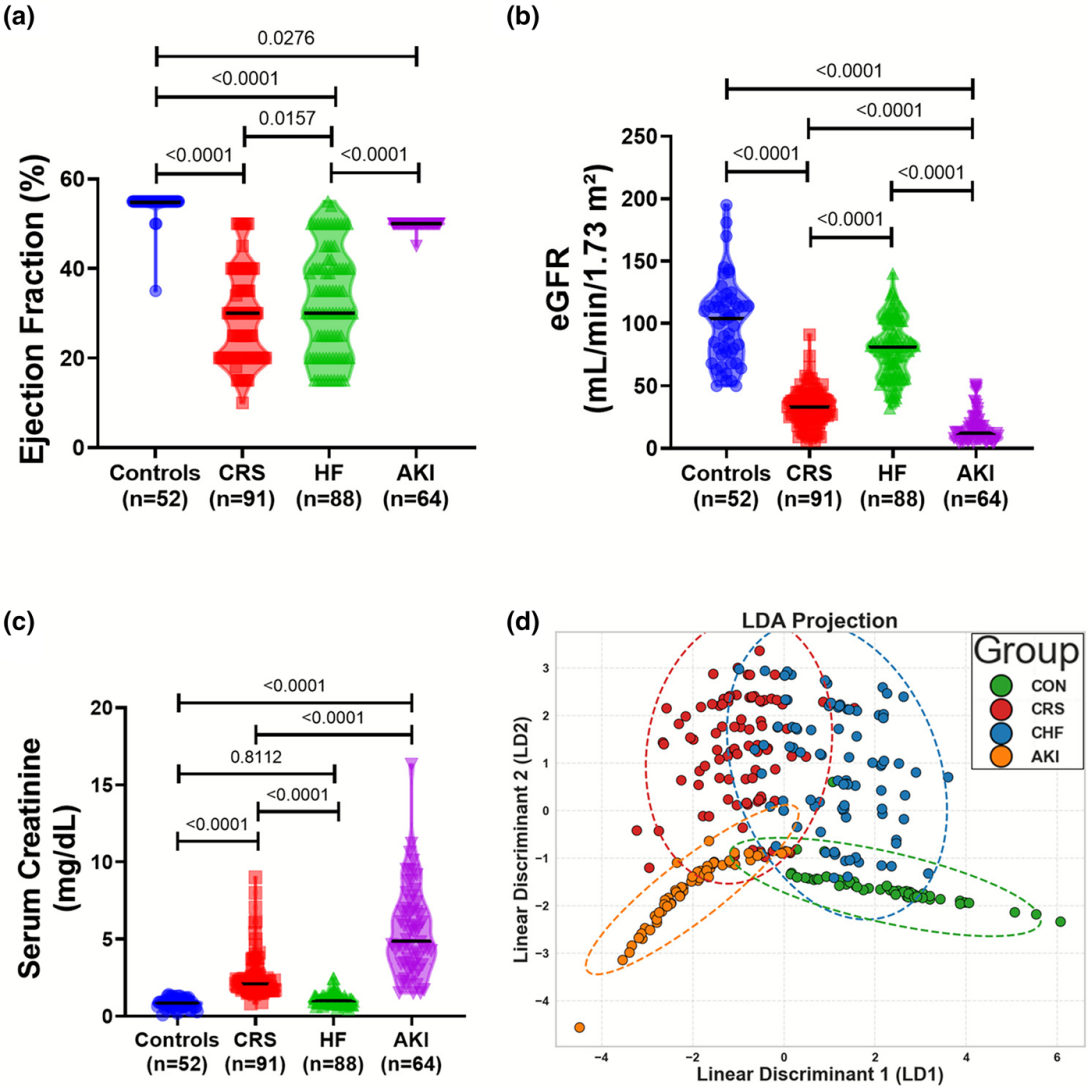
Comparative analysis and clustering of baseline demographics. (a) EF (%), (b) eGFR, and (c) plasma serum creatinine level across groups. (d) Supervised Linear Discriminant Analysis (LDA) projection of patient groups. Statistical analyses were done using one‐way Anova with post‐hoc multiple comparison by Tukey's test.

Our acute CRS cohort groups have distinct pathologies associated with each clinical characteristic of acute CRS, HF, and AKI compared to healthy individuals (Figure 1a–d). EF is a significant surrogate of cardiac function and is used to evaluate cardiac output efficiency. Statistical analysis showed a significant difference in EF across the cohort, with severe compensation in both the CRS group (Group II) and the CHF group (Group III), with a *p*‐value of <0.0001 for both comparisons (Figure 2a). This significant drop in EF is clinically indicative of severe cardiac dysfunction in both HF and acute CRS. In contrast, individuals in the AKI group (Group IV) had a significant difference in EF compared to the healthy group, despite being within the normal EF range (50%–70%), suggesting that the AKI group had normal cardiac function without clinical overlap with CHF or CRS (AHA/ACC/HFSA Guideline for the Management of Heart Failure, 2022 and Heart.org) (Bozkurt, Coats, Tsutsui, Abdelhamid, Adamopoulos, Albert, et al., 2021b; Heidenreich et al., 2022).

The estimated glomerular filtration rate (eGFR) serves as a clinical marker of renal insufficiency. In this cohort, the healthy group demonstrated normal baseline eGFR levels (95.44 ± 32.86 mL/min/1.73 m^2^). As expected, the AKI group presented with severely compromised renal function with significantly reduced eGFR of 12.76 ± 11.88 mL/min/1.73 m^2^ (Figure 2b). Similarly, the CRS group also showed a significantly reduced eGFR (29.05 ± 14.98 mL/min/1.73 m^2^), reflecting impaired renal function superimposed on AHF. The CHF group, compared to the healthy group, had a significant difference in mean eGFR but with no risk criteria for risk of AKI. Further, non‐significant differences in mean sCr level, ruled out risk of AKI pathologies (Figure 2c).

The differences in alcohol consumption and smoking habits between the groups are considered personal and deliberate choices, which might not significantly impact the characterization of the cohort groups. Therefore, these factors were not deemed critical in the analysis of clinical outcomes or the interpretation of the study's findings.

The notable significance, however, lies in the EF levels in CRS. Though it is significantly downplayed, still, the average EF of this group shows considerably impaired functioning than non‐CRS HF patients (Figure 2a). A different pattern can be seen in terms of renal functionalities at the level of sCr and eGFR (Figure 2b,c). CRS patients have relatively better renal functionality than non‐CRS AKI patients.

Further to understand how efficiently EF, eGFR, and sCr can separate and classify the disease groups in a supervised manner, we performed Linear discriminant analysis (LDA). LDA analysis has shown a mean cross‐validated accuracy of 81.36%, indicating a good level of class separation in the cohort (Figure 2f). The acute CRS group has also taken up a distinct space with some overlap with the CHF groups.

Such significant clinical differences in characteristics of acute CRS with allied pathological conditions, CHF and AKI, in both supervised and unsupervised dimensionality reduction methods, signify a unique and multivariate pathophysiology of acute CRS. The possible explanation for this mismatch could be unrealized pathological differences in disease progression yet to be deciphered.

### 8.7 Risk stratification and gender bias in acute CRS

**FIGURE 3:**
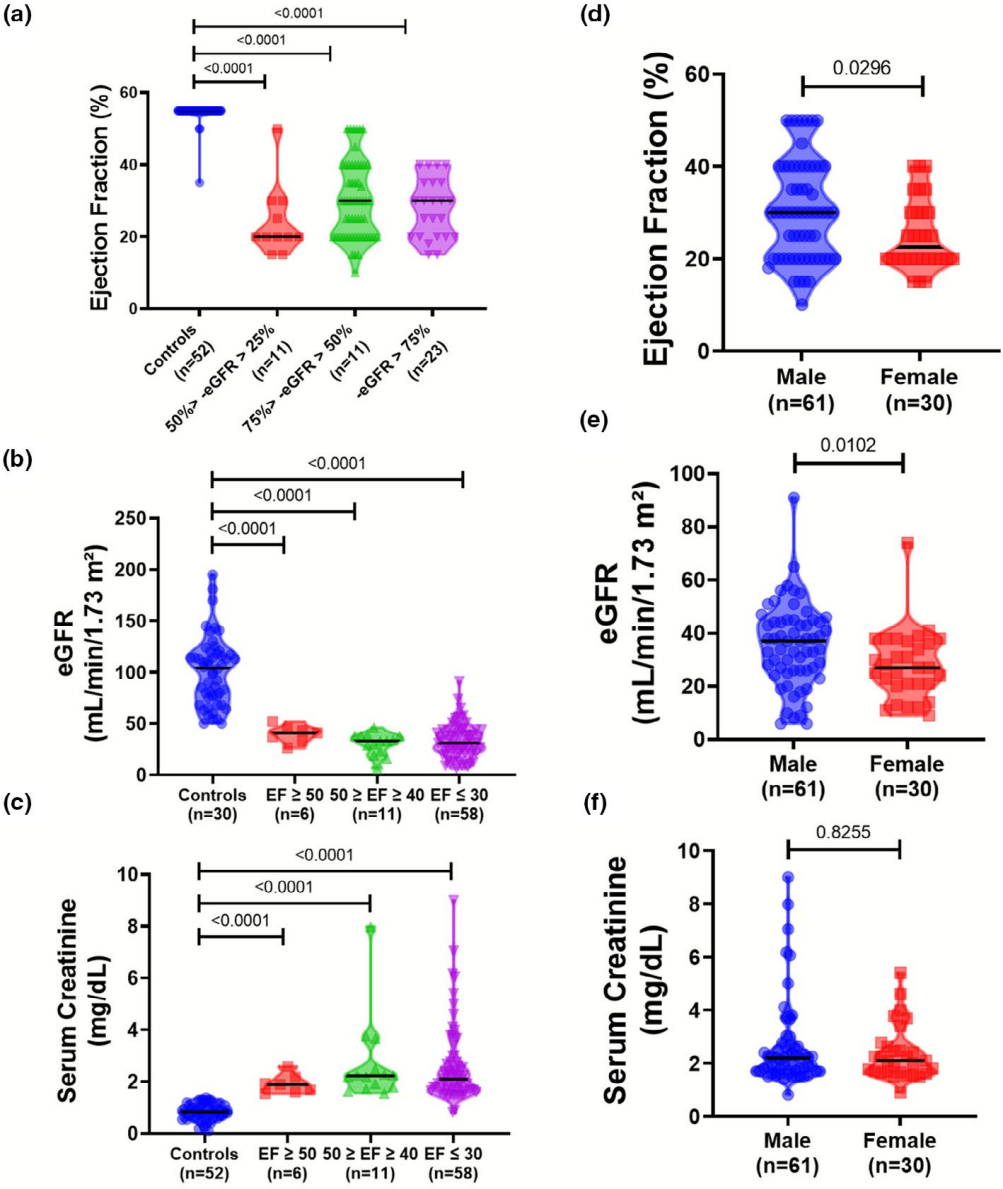
Comparison of disease severity and sex biases in acute CRS. Stratification and association of EF (a), eGFR (b), and sCr (c) in CRS patients' quartiles, classified following *AHA/ACC/HFSA Guideline for the Management of Heart Failure* (b and c) and *RIFLE* (a) guidelines. (a) eGFR: Loss of eGFR, *RIFLE* stratification of CRS type 1: RISK (eGFR loss by >25% and <50%), Injury (eGFR loss by >50% and <75%), and Failure (eGFR loss by >75%). (b and c) Classification of Acute‐CRS according to *AHA/ACC/HFSA Guideline for the Management of Heart Failure*: HFpEF (LVEF ≥50%), HFmrEF (41%–49%), and HFrEF (≤40%). Sex biases in clinical acute CRS patients as observed in clinical parameters like EF (d), eGFR (e), and sCr (f). Statistical analyses were done using one‐way Anova with post‐hoc multiple comparison by Tukey's test and Mann–Whitney *U* test for comparison between two conditions.

HF in clinics are broadly classified into 3 strata–HF with preserved EF (HFpEF), moderately reduced EF (HFmrEF), and reduced EF (HFrEF) based on EF (%) (Table 1) (Bozkurt, Coats, Tsutsui, Abdelhamid, Adamopoulos, Albert, Anker, et al., 2021a). CRS patients with HFpEF have shown similar renal functionalities measured by eGFR and sCr compared to HFmrEF and HFrEF. This suggests that compensated renal function in the pathogenesis of acute CRS is irrespective of cardiac EF (Table 1). To understand how variable grades of renal injury are associated with cardiac EF, we have classified the CRS patients into 3 stages of AKI based on eGFR levels (RIFLE classification, Table 1) (Lopes & Jorge, 2013). Cardiac functionalities didn't vary when classified according to AKI categories (Figure 3a), suggesting that like EF, renal insufficiency also does not vary in acute CRS, in clinics. Furthermore, classified into different quartiles of HF, we found no significant differences in eGFR and sCr between CRS patients with preserved‐normal EF (>50%), mild reduced EF (41%–49%), and reduced EF (<40%) HFpEF and HFmrEF or HFrEF (Figure 3b,c).

Furthermore, we have also tried to understand how sexual differences can affect CRS‐related clinical parameters. HF in a clinical setup has a pronounced bias on male individuals, and females are relatively less affected. However, interestingly the female CRS patients have shown greater cardiorenal dysfunction as suggested by significantly reduced EF (24.13% ± 7.47% compared to 28.49% ± 10.73% in males) and eGFR (25.19 ± 12.73 mL/min/1.73 m^2^ compared to 31.16 ± 15.41 mL/min/1.73 m^2^ in males) in acute CRS (Figure 3d,e). However, the sCr levels didn't differ significantly in females (2.2 ± 1.01 mg/dL) compared to males (2.31 ± 1.6 mg/dL) (Figure 3f).

## 9 DISCUSSION

Given the increasing incidence of global cardiorenal complications, including in India, it is essential to establish diagnostic and prognostic criteria that distinctly classify CRS from non‐CRS AHF and AKI patients (Dutta et al., 2023, 2025; Rangaswami et al., 2019; Zannad & Rossignol, 2018). In this study, we have built a cohort of 295 patients with four different etiologies to delineate only a CRS‐specific understanding of the presently available diagnostic parameters and clinical distinction from the other two overlapping pathologies, that is, CHF and AKI.

Cardiac EF (%) shortening is a clinical sign of reduced cardiac functioning in HF and is significantly lowered in both the CHF and CRS groups (Figure 2a). In a similar trend, decreased eGFR, the conventional clinical parameter used to evaluate clinical renal insufficiency, also shows a significant decrease in both AKI and CRS (Figure 2b). Another traditional clinical parameter for AKI, serum creatinine (sCr), also shows a significant rise in both AKI and CRS patients (Figure 2c). However, eGFR and sCr levels in acute CRS show profound distinction from non‐CRS AKI. The point of ambiguity in clinical characteristics arises from this data, which shows a significant difference in eGFR and sCr between CRS and AKI in this study cohort. Recently emerging circulatory biomarkers of AKI, NGAL, have also shown significant upregulation from that of controls. In CRS, although plasma NGAL levels were significantly up when compared to controls, no significant difference was found in multiple comparisons with one‐way ANOVA in the presence of AKI. Similarly, the emerging AKI‐specific biomarker of renal tubular injury, KIM‐1 (Tutunea‐Fatan et al., 2024), shows higher circulatory detection in 84.6% of the AKI cases recruited. However, only 26.9% of CRS patients showed elevated KIM‐1 in circulation. These further limit the diagnostic ability of gold‐standard and novel AKI biomarkers in distinguishing acute CRS in clinics. This depicts a pressing need for CRS‐specific novel biomarkers, distinguishing acute CRS from HF and AKI.

The acute CRS patients also had significantly reduced EF from non‐CRS HF, which is also an unprecedented observation in clinical settings. Further, the clustering analysis has also made a unique cluster of acute‐CRS patients in both linear and non‐linear mapping (Figure 2d).

This observation indeed suggests that the pathophysiology of acute CRS is fundamentally different from non‐CRS HF or AKI. Acute CRS in clinics is pathologically underlined with reduced cardiac EF and subsequent renal hypoperfusion (Figure 1). The significant difference in clinical demographics in acute CRS versus HF and AKI therein suggests a different pathophysiology of acute CRS. The possible scientific explanation for this observation could be a fundamental involvement of a malfunctioning cardiorenal continuum, which involves elicited cardiorenal crosstalk and activation of hemodynamic mediators. While AHF patients have consistently been shown to develop AKI and/or CKD, not all of them do. Why so? It is a very inquisitive area that needs profound exploration from larger clinical studies as well as basic research to understand the pathophysiological switch for CRS in AHF. Further to understand the disease severity across strata of HF and AKI, we have classified the CRS group into different EF and eGFR quartiles following the Universal Definitions and Classification of HF and AKI (Table 1) (Bozkurt, Coats, Tsutsui, Abdelhamid, Adamopoulos, Albert, Anker, et al., 2021a; Lopes & Jorge, 2013). When segregated into EF quartiles, HFpEF‐CRS patients had similar profound renal dysfunction compared to CRS patients with HFmrEF or HFrEF (Figure 3a). Although the mechanistic understanding of HFpEF is still not completely understood, a pathological connection between the HFpEF heart and the kidney has been previously described to remodel the myocardium differently than HFrEF (Ter Maaten et al., 2016). However, the present data suggest that the preservation of EF in acute CRS affects the renal counterpart similarly to that of reduced EF.

On the other hand, growing pieces of evidence support the manifestation of HF and AKI to be sex‐biased (Golestaneh et al., 2025; Regitz‐Zagrosek & Gebhard, 2023; Soranno et al., 2025). However, the contribution of biological sex is yet to be studied in acute CRS. In this study, we also evaluated EF, eGFR, and sCr levels in the recruited male and female CRS patients. Interestingly, EF and eGFR were significantly decreased in females compared to males. This is not in trend with the present scientific notion of sex biases observed in HF and AKI and warrants more robust investigations in larger cohorts worldwide. However, further mechanistic exploration is needed to draw any concluding remarks about the significance of biological sex in acute CRS pathogenesis. In terms of pathogenesis, the hallmark feature of end‐stage HF and AKI is tissue fibrosis. Fibrotic remodeling pathologically downplays cardiac and renal functions, triggering organ failure. Similarly, in CRS, cardiorenal fibrosis is significantly prevalent and involves upregulation of circulatory fibrotic markers (Dutta et al., 2025; Petra et al., 2021). Altered levels of circulatory factors are a promising field in clinical diagnosis, given that the involvement of intra‐organ crosstalk in CRS is a plausible cause of CRS‐related cardiorenal pathologies.

In summary, in this study of the acute CRS cohort, we found that acute CRS is pathologically distinct from the primary cause, HF, and the secondary cause, AKI, in the clinics.

### 9.1 Future perspective

The complex and multifactorial pathophysiology of acute CRS is clinically divergent in nature, and with increasing prevalence, there is an urgent need for specific diagnostic and prognostic biomarkers and tools. Present clinical means are not specific to acute CRS. Additionally, the parametric differences in eGFR, sCr, and EF in CRS versus AKI and HF highlight the distinct pathophysiology of acute CRS, requiring future investigations.

To address this, an integrative multi‐omics approach, utilizing transcriptomics, proteomics, and lipidomics, is essential to identify specific diagnostic and prognostic markers for acute CRS. A methodical characterization of preclinically and clinically validated novel biomarkers and altered pathways will help in understanding the underlying mechanisms and progression of CRS, thereby contributing to the development of targeted therapies for CRS.

## AUTHOR CONTRIBUTIONS

Abhi Dutta, Sahil Verma, Anupam Mittal, and Trayambak Basak worked on summarizing the main aims and goals of the recently funded Cardio‐renal syndrome (CRS) cohort. Vivek Kumar and Ajay Bahl recruited the study participants for the study cohort. Abhi Dutta and Sahil Verma have prepared the figures and written the manuscript. Trayambak Basak conceptualized the overall structure of the manuscript. All authors have contributed substantial and intellectual contributions to this work.

## FUNDING INFORMATION

This study on cardiorenal syndrome was funded by an ICMR ad hoc project (RFC No. NCD/Ad hoc/207/2022‐2023 dated March 11, 2023).

## CONFLICT OF INTEREST STATEMENT

The authors declare no conflict of interest.

## Data Availability

The source datasets and analyzed data are available on reasonable request.
